# Risk factors and outcomes of bailout procedures during laparoscopic cholecystectomy for acute cholecystitis: the BOLAC study

**DOI:** 10.1007/s00464-026-13040-9

**Published:** 2026-07-15

**Authors:** Marco Ceresoli, Francesca Graziano, Niccolò Allievi, Luca Ansaloni, Matelda Bencini, Ilaria Benzoni, Pietro Bisagni, Luigi Boccia, Bruno Branciforte, Diletta Cassini, Daniele Del Fabbro, Michele Fogliata, Marco Garatti, Luca Ghirardelli, Antonio Guadalaxara, Guglielmo Guerrazzi, Marco Lotti, Diego Mariani, Michele Marini, Enrico Pinotti, Andrea Pisani Ceretti, Eliana Spada, Andrea Spota, Nereo Vettoretto, Luca Nespoli, Study Collaborative Group, Chiara Donati, Camilla Dossi, Michele Rossi, Marco Cereda, Stefania Galimberti, Ileana Tiraboschi, Francesco Salvetti, Luciano Digiesi, Cristina Lo Presti, Rita Stanco, Simone Frassini, Paola Fugazzola, Carlo Stracqualursi, Francesco Roscio, Gianluca Baiocchi, Valentina Ricci, Matteo Capuzzo, Michele Ballabio, Ludovica Gibelli, Mariella Alloggio, Hayato Kurihara, Giulia Germiniasi, Giuseppe Faillace, Emilia Masci, Marco Cantamessa, Lorenzo Rosa, Mauro Zago, Fulvio Tagliabue, Giuseppe Zimmitti, Diego Rigosa, Enrico Oriani, Natasa Samardzic, Pierpaolo Mariani, Agnese Serra, Luna Cariola, Camilla Sala, Paolo Pizzini, Gianandrea Baldazzi, Anna Zanaga, Elisa Rondi, Irene Ruggirello, Giuseppe Cordaro, Giuseppe Pizzardi, Nirvana Maroni, Caterina Froiio, Andrea Caramma, Ildo Scandroglio, Stefania Cimbanassi, Sophia Costacurta, Alessandro Asaro, Claudio Guarneri, Arianna Dendena

**Affiliations:** 1 School of Medicine and Surgery, University of Milano-Bicocca, Milan, Italy; 2 General and Emergency Surgery, Fondazione IRCCS San Gerardo Dei Tintori, Monza, Italy; 3 Biostatistics and Clinical Epidemiology, Fondazione IRCCS San Gerardo Dei Tintori, Monza, Italy; 4 General and Emergency Surgery, ASST Papa Giovanni XXIII, Bergamo, Italy; 5 General and Emergency Surgery, Fondazione IRCCS San Matteo, Pavia, Italy; 6 General Surgery, ASST Valleolona, Ospedale Sant’Antonio Abate, Gallarate, Italy; 7 General Surgery, ASST Cremona, Cremona, Italy; 8 General and Emergency Surgery, ASST Lodi, Lodi, Italy; 9 General and Emergency Surgery, ASST Mantova, Mantua, Italy; 10 Emergency Surgery, Fondazione IRCCS Ca’ Granda Ospedale Maggiore Policlinico, Milan, Italy; 11 Emergency and General Surgery, ASST Nord-Milano, Sesto San Giovanni Hospital, Sesto San Giovanni, Italy; 12 Emergency and Trauma Surgery Unit, IRCCS Humanitas Research Hospital, Rozzano, Italy; 13 Emergency and General Surgery, ASST Lecco, Lecco, Italy; 14 General, Oncologic and Robotic Surgery, Fondazione Poliambulanza, Brescia, Italy; 15 General and Emergency Surgery Department, San Raffaele Scientific Institute, Milan, Italy; 16 General Surgery, ASST Bergamo-Est, Ospedale Di Lovere, Lovere, Italy; 17 General Surgery, ASST Bergamo-Est, Ospedale Di Alzano Lombardo, Alzano Lombardo, Italy; 18 General and Emergency Surgery, ASST Fatebenefratelli-Sacco, Ospedale Fatebenefratelli, Milan, Italy; 19 General and Emergency Surgery, ASST Ovest-Milano, Ospedale Di Legnano, Legnano, Italy; 20 General and Emergency Surgery, ASST Bergamo-Est, Ospedale Di Seriate, Seriate, Italy; 21 General Surgery, Policlinico San Pietro, Ponte San Pietro, Italy; 22 Emergency and General Surgery, ASST Nord-Milano, Bassini Hospital, Cinisello Balsamo, Italy; 23 General Surgery, ASST Valleolona, Ospedale Di Busto Arsizio, Busto Arsizio, Italy; 24 Emergency and Trauma Surgery, ASST GOM Niguarda, Milan, Italy; 25 Department of Surgery, ASST Spedali Civili Di Brescia, Montichiari, Italy

**Keywords:** Bailout procedure, Acute cholecystitis, Laparoscopic cholecystectomy, Subtotal cholecystectomy, Bile duct injury

## Abstract

**Background:**

Bailout procedures during laparoscopic cholecystectomy are employed to prevent major complications. However, their incidence, determinants, and clinical impact remain incompletely defined in large multicenter settings. This study aimed to assess the incidence of bailout procedures during laparoscopic cholecystectomy for acute cholecystitis, identify associated risk factors, and evaluate postoperative outcomes.

**Methods:**

A retrospective multicenter cohort study was conducted across 22 hospitals in Northern Italy, including consecutive adult patients undergoing laparoscopic cholecystectomy for acute calculous cholecystitis between January 2023 and December 2024. Bailout procedures were defined as any deviation from standard laparoscopic cholecystectomy, including subtotal cholecystectomy or conversion to open surgery. Multivariable logistic regression was used to identify independent predictors. Inter-center variability was assessed using the median odds ratio (MOR).

**Results:**

Among 1805 patients, bailout procedures were required in 176 cases (9.8%). The most common strategies were open conversion (4.7%) and laparoscopic subtotal cholecystectomy (3.3%). Failure to achieve the Critical View of Safety was the leading indication (54.2%). Independent predictors of bailout included intramural abscess (OR = 3.25), male sex (OR = 1.74), peritoneal collection (OR = 1.77), and surgical delay > 7 days (OR = 1.85). Significant inter-center variability was observed (MOR = 2.42). Bailout procedures were associated with worse outcomes, including longer hospital stay (11.8 vs. 6.7 days), higher severe complication rates (4.5 vs. 1.6%), increased bile leaks (10.8 vs. 2.3%), and higher readmission rates (6.8 vs. 2.3%).

**Conclusions:**

Bailout procedures are required in approximately 10% of LCs for AC and are strongly associated with markers of advanced inflammation and delayed surgery. Their use reflects both disease severity and institutional practice variability and is associated with increased postoperative morbidity. Early intervention and improved risk stratification may optimize surgical planning and outcomes.

**Supplementary Information:**

The online version contains supplementary material available at https://doi.org/10.1007/s00464-026-13040-9.

Acute cholecystitis (AC) remains one of the most frequent indications for emergency surgical consultation worldwide. Over the past few decades, the therapeutic gold standard has shifted decisively toward early laparoscopic cholecystectomy (LC) [1–4]. However, the “difficult gallbladder”—characterized by intense local inflammation, gangrenous changes, or dense adhesions—continues to challenge even the most experienced surgical teams [5].

The primary goal during a difficult LC is the prevention of catastrophic complications, such as bile duct injury (BDI). To mitigate this risk, the “Culture of Safety in Cholecystectomy” emphasizes the achievement of the Critical View of Safety (CVS) as a non-negotiable prerequisite for clipping and dividing the cystic duct and artery [6–10]. When severe inflammation or distorted anatomy precludes the safe attainment of CVS, the surgeon must pivot. This pivot, often referred to as a “bailout procedure,” encompasses a range of techniques including laparoscopic or open subtotal cholecystectomy (reconstructing or fenestrated) and traditional conversion to open total cholecystectomy [11].

While bailout procedures are conceptually designed to protect the patient, they are not without their own inherent risks. Recent literature suggests that while these techniques effectively reduce the incidence of BDI, they may be associated with increased rates of postoperative bile leaks, surgical site infections, and prolonged hospitalization [12, 13]. Furthermore, the decision to “bail out” is often subjective, potentially influenced not only by patient-specific pathology but also by surgeon experience, sub-specialization, and institutional culture.

The management of acute cholecystitis is commonly distributed across a wide variety of hospital settings, from high-volume academic centers to smaller community hospitals [14]. Despite the frequency of these procedures, there is a lack of large-scale, multicenter data characterizing the incidence and specific risk factors that lead to bailout maneuvers and the subsequent impact on patient recovery.

The BOLAC (BailOut procedures during Laparoscopic cholecystectomy for Acute Cholecystitis) study was designed to address these gaps. By leveraging a large, multicenter retrospective cohort from 22 hospitals in Northern Italy, this study aims to define the incidence of bailout procedures, identify independent preoperative and intraoperative predictors of their use, and evaluate the associated postoperative outcomes.

## Methods

This is a multicenter retrospective observational study conducted between January 1, 2023, and December 31, 2024, across 22 hospitals in the Lombardia Region, Northern Italy. The study protocol was endorsed by the Società Lombarda di Chirurgia and was approved by the local Ethic committee. The study included all consecutive adult patients treated with laparoscopic cholecystectomy for acute calculous cholecystitis during the index admission.

The primary endpoint of the study was the incidence of need for bailout procedures. Secondary endpoint included risk factors for bailout procedures and postoperative outcomes.

Inclusion criteria were: age > 18 years, a confirmed clinical, biochemical, and radiological diagnosis of acute calculous cholecystitis according to TG18 diagnostic criteria [15], urgent or unplanned hospital admission and same admission emergency or urgent laparoscopic cholecystectomy. Exclusion criteria included elective cholecystectomy, delayed cholecystectomy after conservative treatment (further hospitalization), those with alternative diagnoses, and those undergoing primary open surgery. Data were retrospectively collected from institutional databases and recorded anonymously in a dedicated electronic dataset.

Collected variables included demographic characteristics, comorbidity burden assessed using the Charlson Comorbidity Index. Severe sepsis and septic shock were defined in accordance with international consensus criteria (Sepsis 3). Operative variables included timing of surgery calculated from the patient-reported date of symptom onset as documented in the medical history at admission, timing of the day and calendar period, operative time, identification of the critical view of safety, and the use of bailout procedures, with reason for bailout. Postoperative complications occurring within 30 days of surgery were systematically recorded and graded using the Clavien-Dindo classification system, with major morbidity defined as a Clavien-Dindo > II.

Bailout procedures were defined as any deviation from standard laparoscopic cholecystectomy for any reason, according to the definition provided by Strasberg et al. and they include: laparoscopic subtotal cholecystectomy (reconstructing or fenestrated), conversion to open surgery with complete cholecystectomy or subtotal open cholecystectomy (reconstructing or fenestrated) [11].

For each surgical procedure were recorded the age and the sub-specialty of the most experienced surgeon of the operating equipe. Operating surgeons were arbitrary classified as colorectal (> 50% of working time dedicated to elective colorectal surgery, > 30 laparoscopic colorectal resection/year), acute care (> 50% of working time dedicated to emergency general surgery and trauma), HPB (> 50% of working time dedicated to major hepatobiliary surgery, > 10 major resection/year) or other, since in Italy there’s no structured fellowships or recognized under-specialization.

Each participating center’s characteristics were also collected, including availability of advanced hepatobiliary surgery service, interventional radiology and interventional endoscopy. Postoperative outcomes included intensive care unit admission, postoperative complications, bile leak, bile duct injury, reoperation, and length of hospital stay.

### Statistical analysis

Continuous variables were expressed as mean and standard deviation (SD) or median and interquartile range (IQR), depending on distribution, while categorical variables were expressed as absolute number and percentage. Univariate analysis was performed using appropriate statistical tests depending on variable distribution. Variables found to be significant were included in multivariate logistic regression analysis to identify independent predictors of bailout procedures.

Between-center variability in bailout procedure was quantified using the median odds ratio (MOR), derived from unadjusted and adjusted generalized linear mixed modeling, with center included as random effect. Models were also adjusted for the case-mix; specifically, they were adjusted for age, sex, Charlson Comorbidity (CC) Index, intramural abscesses, peritoneal collections, and time from admission to surgery. The MOR represents the odds of undergoing bailout procedure when comparison two randomly selected centers for patients with the same covariates. MOR greater than one denotes higher variability among centers. Statistical significance was defined as a p-value less than 0.05. All analyses were conducted with R (4.5.1).

## Results

### Study population

During the study period, a total of 1805 patients undergoing laparoscopic cholecystectomy for acute calculous cholecystitis were included. The mean age was 62.20 years (± 16.24) and a slight male predominance (57.2%). The cohort had an average BMI of 27.44 kg/m^2^ and a mean Charlson Comorbidity Index (CCI) of 2.52.

At presentation the median WBC were 13.00 *10^9^/L (IQR = 10.10–16.51) and median CRP level was 65.60 mg/L (IQR = 15.12–190.60). The most commonly used imaging modality were ultrasound (76.8%) and CT scan (43.0%), while 18.8% of the patients required second-level imaging (EUS or MRCP) due to suspected common bile duct stones. The mean time to surgery was 2.96 (± 4.70) days. Table 1 shows patients’ characteristics in detail.

**Table 1:** Patients’ characteristics overall and stratified by type of procedures

	Overall	Laparoscopic cholecystectomy	Bailout procedures (overall)	*p*-value
	1805	1629	176	
Age (mean (SD))	62.20 (16.24)	61.57 (16.38)	68.01 (13.57)	< 0.001
Sex M (%)	1033 (57.2)	911 (55.9)	122 (69.3)	0.003
BMI (mean (SD))	27.44 (4.83)	27.39 (4.81)	27.85 (4.95)	0.353
Charlson Comorbidity Index (mean (SD))	2.52 (2.20)	2.42 (2.15)	3.43 (2.48)	< 0.001
Septic shock at presentation (%)	33 (1.8)	28 (1.7)	5 (2.8)	0.449
Severe sepsis at presentation (%)	150 (8.3)	128 (7.9)	22 (12.6)	0.046
WBC (median [IQR])	13.00 [10.10, 16.51]	13.00 [10.00, 16.26]	13.95 [10.57, 17.90]	0.017
CRP (mg/L) (median [IQR])	65.60 [15.12, 190.60]	60.00 [14.00, 175.00]	134.50 [28.07, 238.70]	< 0.001
PCT (median [IQR])	0.80 [0.14, 3.07]	0.76 [0.12, 2.78]	1.34 [0.49, 10.85]	0.006
Serum bilirubin (median [IQR])	1.10 [0.70, 1.98]	1.10 [0.70, 1.98]	1.21 [0.80, 2.02]	0.060
US (%)	1386 (76.8)	1267 (77.8)	119 (67.6)	0.003
US wall thickness (mm) (mean (SD))	6.17 (2.77)	6.16 (2.81)	6.25 (2.43)	0.784
Intramural abscesses (%)	143 (8.0)	110 (6.8)	33 (19.1)	< 0.001
Peritoneal collection (%)	253 (14.1)	209 (12.9)	44 (25.3)	< 0.001
CT scan (%)	774 (43.0)	671 (41.3)	103 (58.5)	< 0.001
Preoperative second-level imaging for suspected CBD stones (%)	339 (18.8)	299 (18.4)	40 (22.7)	0.192
Preoperative second-level imaging performed (EUS/MRCP) (%)				
EUS	169 (48.3)	150 (48.4)	19 (47.5)	1.000
MRCP	181 (51.7)	160 (51.6)	21 (52.5)	
Preoperative ERCP (%)	171 (9.5)	148 (9.1)	23 (13.1)	0.116
Preoperative percutaneous cholecystostomy (%)	32 (1.8)	24 (1.5)	8 (4.5)	0.008
Time of the day (%)				
Afternoon	1028 (57.0)	917 (56.4)	111 (63.1)	0.001
Morning	645 (35.8)	601 (37.0)	44 (25.0)	
Night	129 (7.2)	108 (6.6)	21 (11.9)	
Calendar period (%)				
Week	1454 (81.2)	1320 (81.7)	134 (76.6)	0.030
Weekend	311 (17.4)	276 (17.1)	35 (20.0)	
Holiday	25 (1.4)	19 (1.2)	6 (3.4)	
Timing of surgery (days) (mean (SD))	2.96 (4.70)	2.88 (4.60)	3.72 (5.54)	0.025
Bailout procedure	176 (9.8)	0	176 (100)	
Bailout procedure detail				
Laparoscopic subtotal reconstructing			59 (3.3)	
Laparoscopic subtotal fenestrated			9 (0.5)	
Open Cholecystectomy			85 (4.7)	
Open subtotal reconstructing			21 (1.2)	
Open subtotal fenestrated			2 (0.1)	
Reason for bailout procedure (%)				
CVS not achieved			85 (54.2)	
Adherences			46 (29.7)	
Bleeding			9 (5.8)	
CBD injury/bile leak			2 (1.3)	
Other			14 (9.0)	
Sub-speciality of the operator				
Acute care	940 (52.1)	844 (51.8)	96 (54.5)	0.378
Colorectal	178 (9.9)	164 (10.1)	14 (8.0)	
HPB	228 (12.6)	212 (13.0)	16 (9.1)	
Other	459 (25.4)	409 (25.1)	50 (28.4)	
Age of the operator (mean (SD))	47.22 (9.44)	47.05 (9.30)	48.76 (10.54)	0.022
ICG adopted (%)	376 (20.8)	345 (21.2)	31 (17.6)	0.313

*CBD* common bile duct, *CVS* critical view of safety; *HB* hepatobiliary, *ICG* indocyanine green fluorescence, *ACC* acute calculous cholecystitis

**Table 2:** Hospital characteristics

	Level	Overall
*n*		22
Number of cholecysetectomies for ACC (mean (SD))		82.05 (45.36)
Hospital type (%)	Private	4 (18.2)
	Public	18 (81.8)
Number of beds (mean (SD))		25.23 (6.56)
Hospital level (%)	1	6 (27.3)
	2	5 (22.7)
	3	11 (50.0)
Expertise in HB surgery (%)		8 (36.4)
Interventional endoscopy available (%)		16 (72.7)
Interventional radiology available (%)		14 (63.6)
ICG available (%)		14 (63.63)

*HB* hepatobiliary, *ICG* indocyanine green fluorescence, *ACC* acute calculous cholecystitis

### Bailout procedures

**Fig. 1:**
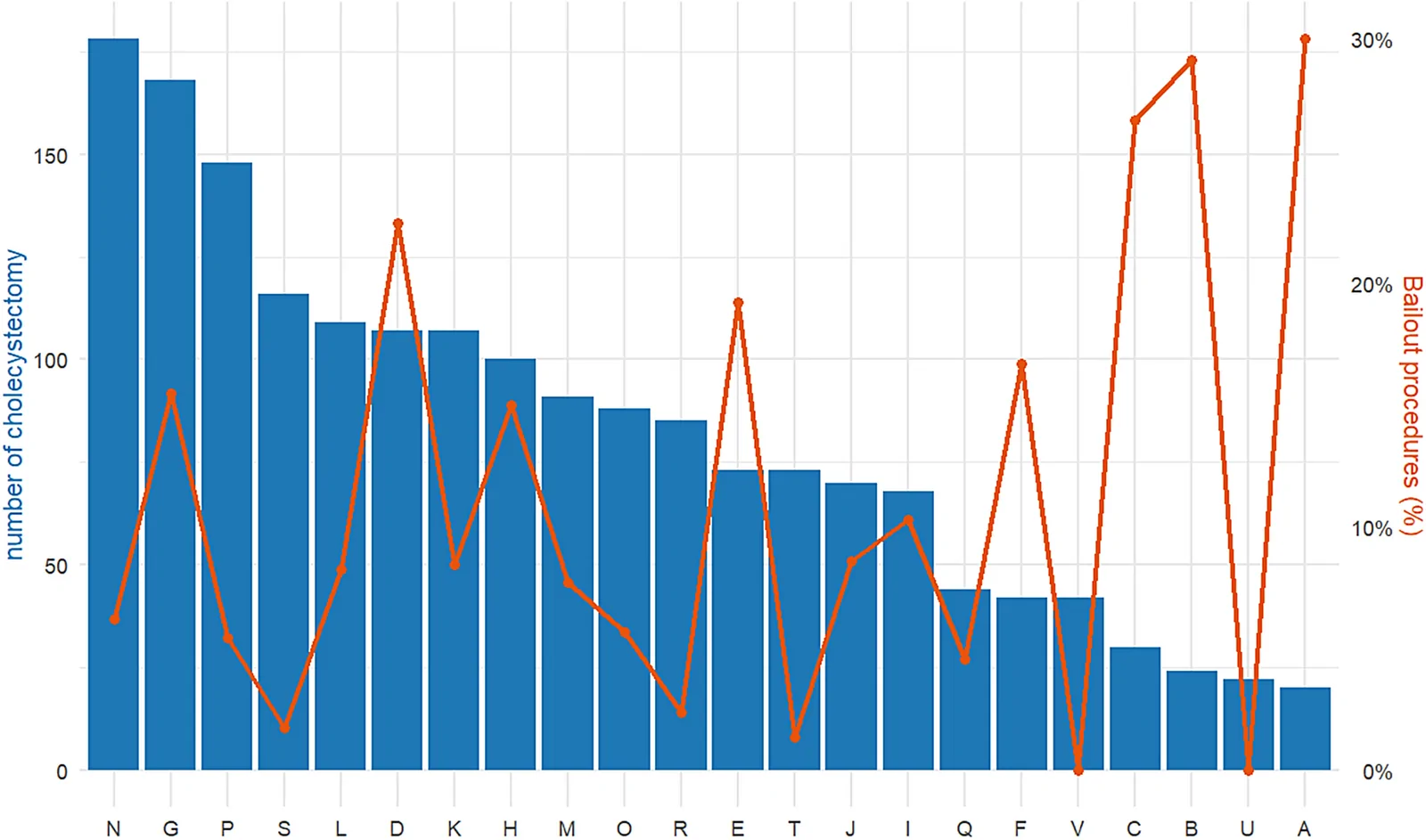
Volume of cholecystectomies and proportion of bailout procedures across participating centers

Bailout procedures were required in 176 cases (9.8%). The most frequent adopted bailout techniques were open cholecystectomy (*n* = 85, 4.7%) and laparoscopic subtotal reconstructing cholecystectomy (*n* = 59, 3.3%), while fenestrated techniques and open subtotal procedures were less commonly used. The primary reason for bailout was the inability to safely achieve the Critical View of Safety (CVS) (54.2%), followed by the presence of adhesions (29.7%) and intraoperative bleeding (5.8%). The distribution of bailout procedure among the total number of cholecystectomies is shown in Fig. 1. Supplementary Table S1 shows detailed clinical data divided for each bailout procedure.

When comparing the two groups (Table 1), patients requiring a bailout procedure were significantly older (68.01 vs. 61.57 years; *p* < 0.001) and more frequently male (69.3% vs. 55.9%; *p* = 0.003). They also had a higher comorbidity burden (mean CCI 3.43 vs. 2.42; *p* < 0.001) and more pronounced systemic inflammation (CRP 134.50 vs. 60.00 mg/L; *p* < 0.001). Bailout procedures occurred more frequently during night shifts (11.9% vs. 6.6%; *p* = 0.001). Surgeon experience and subspeciality did not differ significantly between the two groups.

At multivariable logistic regression analysis the presence of an intramural abscess (OR = 3.25; 95% CI: 1.91–5.52; *p* < 0.001), male sex (OR = 1.74; 95% CI: 1.22–2.47; *p* = 0.002), peritoneal collection (OR = 1.77; 95% CI: 1.13–2.79; *p* = 0.013), and surgical timing exceeding 7 days from symptom onset (OR = 1.85; 95% CI: 1.08–3.14; *p* = 0.024) were identified as independent predictors for the requirement of a bailout procedure. Detailed results are reported in Table 3.

**Table 3:** Results from logistic regression model

Predictors	OR	95% CI	*p*-value
Age	1.010	(0.993–1028)	0.231
Sex M	1.736	(1220–2471)	0.002
CC index	1.089	(0.974–1219)	0.135
Intramural abscess	3.250	(1914–5517)	< 0.001
Peritoneal collection	1.773	(1126–2793)	0.013
Timing > 3 days < 7 days	1.305	(0.880–1935)	0.186
Timing > 7 days	1.845	(1083–3143)	0.024
Hospital level 2	1.092	(0.306–3901)	0.892
Hospital level 3	0.664	(0.232–1898)	0.444

### Centers and centers variability

A total of 22 hospitals participated in the study, with a mean number of cholecystectomies for acute cholecystitis of 82.05 (± 45.36) per center. Most hospitals were public (81.8%), while 18.2% were private institutions. Advanced hepatobiliary surgical expertise was available in 36.4% of centers, interventional endoscopy in 72.7%, and interventional radiology in 63.6%. Indocyanine green (ICG) fluorescence imaging was available in 63.6% of centers. Further details are reported in Table 2.

**Fig. 2:**
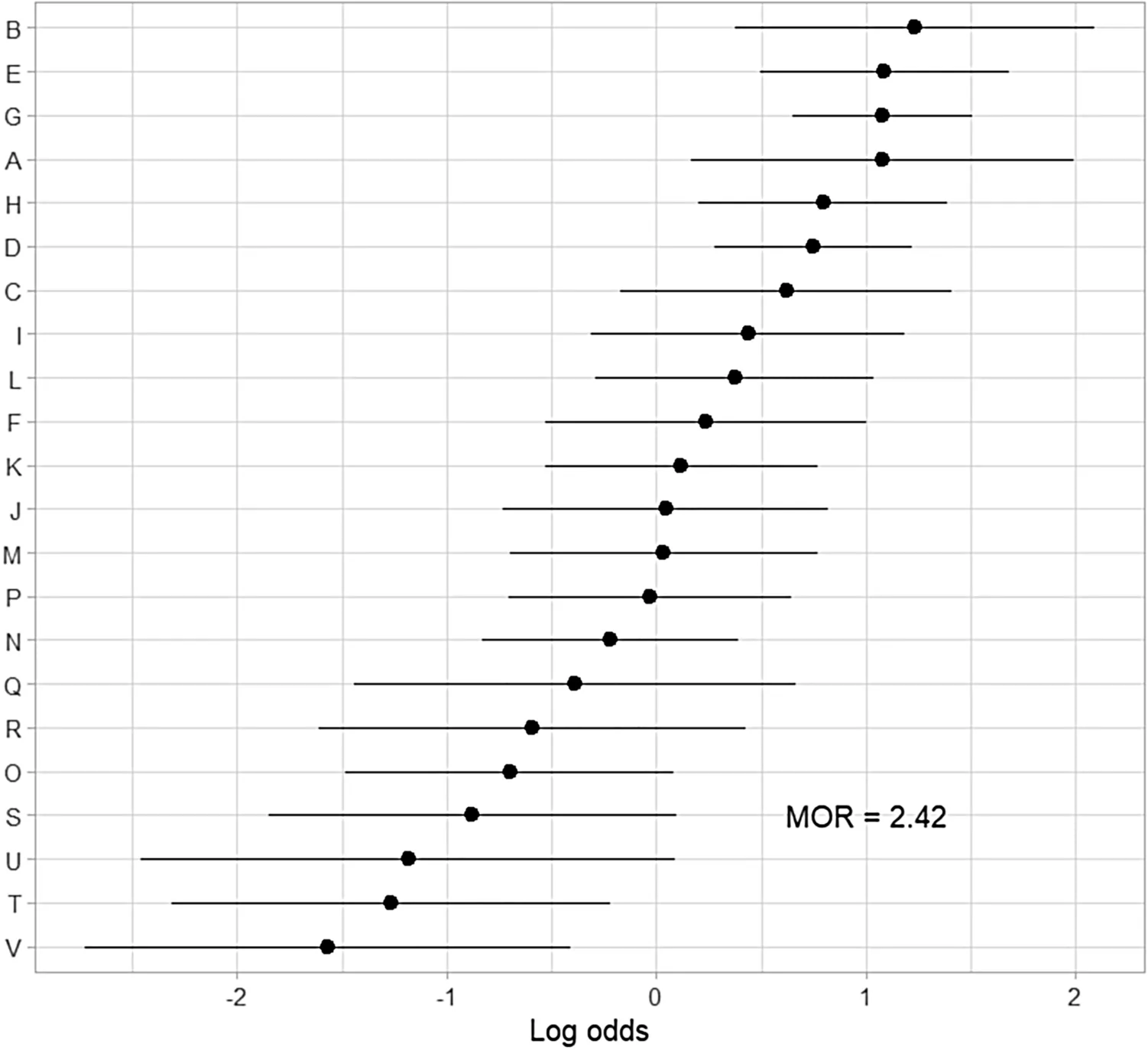
Between-center variability in the use of bailout procedures. Caterpillar plot showing center-specific effects (log odds) for the probability of requiring a bailout procedure across participating hospitals. Each point represents the estimated effect for an individual center, while horizontal lines indicate the corresponding 95% confidence intervals

A significant inter-center variability in the use of bailout procedures was observed across the participating hospitals, with a high median odds ratio (MOR) from adjusted model of 2.42, as shown in the caterpillar plot (Fig. 2).

### Outcomes

Patients requiring a bailout procedure experienced significantly worse clinical outcomes compared to those who underwent standard laparoscopic completion. The length of stay (11.81 vs. 6.71 days; *p* < 0.001) and the incidence of severe complications (4.5 vs. 1.6%; *p* = 0.015) were higher in the bailout group. Patients with bailout procedure had higher bile leaks (10.8 vs. 2.3%; *p* < 0.001) and higher 30-day readmission rate (6.8 vs. 2.3%; *p* = 0.001). Detailed results are shown in Table 4.

**Table 4:** Clinical outcomes, overall and by procedures

	Level	Overall	Laparoscopic cholecystectomy	Bailout procedures	*p*
*n*		1805	1629	176	
Mortality (%)		6 (0.3)	3 (0.2)	3 (1.7)	< 0.001
Morbidity, any (%)		471 (26.1)	398 (24.4)	73 (41.5)	< 0.001
Complications grade (%)	0	1334 (73.9)	1231 (75.6)	103 (58.5)	< 0.001
	I	256 (14.2)	241 (14.8)	15 (8.5)	
	II	108 (6.0)	85 (5.2)	23 (13.1)	
	IIIa	72 (4.0)	45 (2.8)	27 (15.3)	
	IIIb	17 (0.9)	14 (0.9)	3 (1.7)	
	IV	12 (0.7)	10 (0.6)	2 (1.1)	
	V	6 (0.3)	3 (0.2)	3 (1.7)	
Length of stay (mean (SD))		7.21 (7.55)	6.71 (7.04)	11.81 (10.12)	< 0.001
Reoperation (%)		18 (1.0)	14 (0.9)	4 (2.3)	0.135
Severe complications (%)		35 (1.9)	27 (1.6)	8 (4.5)	0.013
Bile leak (%)		57 (3.2)	38 (2.3)	19 (10.8)	< 0.001
CBD injury (%)		11 (0.6)	5 (0.3)	6 (3.4)	< 0.001
Superficial SSI (%)		22 (1.2)	16 (1.0)	6 (3.4)	0.010
Deep SSI (abscess/collection) (%)		59 (3.3)	44 (2.7)	15 (8.5)	< 0.001
Respiratory infections (%)		40 (2.2)	27 (1.7)	13 (7.4)	< 0.001
Renal failure (%)		33 (1.8)	21 (1.3)	12 (6.8)	< 0.001
Urinary infections (%)		8 (0.4)	7 (0.4)	1 (0.6)	0.694
Cardio complications (%)		39 (2.2)	29 (1.8)	10 (5.7)	0.009
Readmission within 30 days (%)		49 (2.7)	37 (2.3)	12 (6.8)	0.005

*CBD* common bile duct, *SSI* surgical site infection

Supplementary Table 2 presents outcomes stratified by each bailout procedure: laparoscopic subtotal cholecystectomy showed the highest rate of bile leaks (14.7%) and the longest mean length of hospital stay (12.34 days), while open cholecystectomy had the highest overall morbidity (45.9%).

## Discussion

In this multicenter cohort of 1805 patients undergoing laparoscopic cholecystectomy for acute calculous cholecystitis, bailout procedures were required in 9.8% of patients. Open conversion and laparoscopic subtotal reconstructing cholecystectomy represented the most commonly adopted strategies, with failure to achieve the Critical View of Safety being the leading intraoperative indication. Multivariable analysis identified intramural abscess, peritoneal fluid collection, male sex, and delayed surgical timing beyond seven days from symptom onset as independent predictors of bailout procedures. The need for a bailout procedure was associated with worse postoperative outcomes, including longer hospital stay, higher rates of severe complications, increased incidence of bile leaks, and greater 30-day readmission rates.

This study represents one of the largest multicenter cohorts focused specifically on bailout procedures during laparoscopic cholecystectomy for acute calculous cholecystitis. Our findings are consistent with the rates of bailout procedures reported in the current literature on difficult cholecystectomy [16, 17]. Previous multicenter and registry-based studies have generally described bailout rates ranging between 5 and 15% in patients undergoing laparoscopic cholecystectomy for acute cholecystitis. Similarly, conversion to open surgery and subtotal cholecystectomy are consistently described as the most frequently adopted bailout strategies when safe completion of the procedure is not feasible. In line with previous reports, male sex, advanced age, and markers of severe local inflammation were associated with a higher likelihood of requiring a bailout procedure, reflecting the well-known relationship between disease severity and operative difficulty. The identification of delayed surgery and specific imaging findings such as intramural abscess or peritoneal collection as independent predictors confirm that advanced inflammatory changes significantly increase the technical complexity of cholecystectomy.

The predictors identified in our multivariable analysis are largely consistent with previously reported determinants of difficult cholecystectomy as the Nassar Scale [18]. In particular, the association between male sex and the need for a bailout procedure observed in our study has been repeatedly reported in the literature and is considered a surrogate marker of more advanced inflammatory disease. Likewise, the presence of intramural abscess and peritoneal fluid collection reflects severe local inflammation and distorted anatomy. Furthermore, the strong predictive value of intramural abscess (OR = 3.25) and peritoneal collection underscores the vital role of high-quality preoperative imaging. Detailed imaging acts as a “roadmap,” allowing for proactive surgical planning and the early involvement of appropriate expertise.

Interestingly, our multivariable analysis confirms a shift in the traditional surgical window (the “72-h rule”), showing that a surgical timing exceeding seven days from symptom onset was independently associated with an increased need for a bailout procedure (OR = 1.85) [19]. However, this finding must be interpreted with caution and should not be viewed as a direct causal relationship. First, the 'time from symptom onset' is inherently limited by the imprecision of patient recall at admission. Second, a major selection bias must be acknowledged: patients undergoing surgery after the seven-day mark in a real-world emergency setting frequently represent cases of failed initial conservative management.

An intriguing aspect of our data is the influence of “when” and “where the surgery occurs. Bailout procedures were significantly more frequent during night shifts and weekends. In these settings, a “safety-first” approach prevails more commonly; a lower threshold for bailout procedures is a pragmatic response to the reduced availability of senior staff or specialized surgical support during off-hours. Interestingly, the hospital’s level (accreditation or size) did not significantly impact the requirement for a bailout, suggesting that the decision to pivot is driven more by pathology, immediate resources than by institutional tier.

One of the most striking and consequential findings of the BOLAC study is the substantial inter-center variability in the utilization of bailout procedures, characterized by a Median Odds Ratio (MOR) of 2.42. This marked variability remained after adjustment for differences in patient case mix, indicating that factors related to individual centers may significantly influence surgical practice. As illustrated in Fig. 1, also a considerable heterogeneity was observed in treatment strategies across participating centers. The wide variation in the number of patients undergoing early laparoscopic cholecystectomy likely reflects not only differences in hospital volume and level of care, but also divergent management approaches, with some centers favoring initial conservative treatment followed by delayed surgery. This high degree of heterogeneity strongly indicates that the decision to ‘bail out’ is not dictated solely by objective, patient-specific pathology, but is highly sensitive to institutional cultures, localized practice styles, and individual surgeon risk tolerance. Currently, there is a distinct lack of standardized, universally accepted intraoperative criteria or objective time-based rules (e.g., a mandatory threshold for conversion after a specific duration of unsuccessful dissection) across the participating network. Consequently, the decision to pivot remains highly subjective. In real-world emergency settings, this subjectivity is further compounded by localized resource constraints and structural variations, as evidenced by the significantly higher rate of bailout maneuvers witnessed during night shifts and weekends when senior or more experienced support may be reduced. The wide variations in both the incidence and selection of bailout techniques documented in our cohort underscore an urgent need to transition from subjective surgeon intuition to standardized, criteria-driven decision algorithms. Incorporating objective anatomical checklists and explicit difficulty grading scales into institutional guidelines could significantly harmonize surgical practice, reduce uncertified inter-center variance, and ultimately optimize patient safety.

Choosing a bailout strategy is not without consequences. While these maneuvers are protective against catastrophic complications such as common bile duct injuries, they are associated with a significant safety-morbidity trade-off. Subtotal cholecystectomies, particularly laparoscopic ones, showed high rates of biliary leaks (14.7%), frequently requiring secondary endoscopic or radiological interventions and leading to nearly doubled hospital stays.

It is important to interpret the worse postoperative outcomes observed in the bailout group with caution, as this association is inherently subject to confounding by indication. Patients requiring a bailout procedure were significantly older, carried a higher comorbidity burden and exhibited higher systemic and local inflammation at presentation. Therefore, these unfavorable outcomes—such as prolonged hospitalization and higher complication rates—are highly reflective also of baseline disease severity and hostile local anatomy rather than being only causally driven by the bailout strategies themselves.

Crucially, the higher incidence of CBD injury observed in the open cholecystectomy group (5.9%) must be interpreted correctly. Rather than being a failure of the open approach itself, some of these injuries should be interpreted as the cause of the bailout procedure rather than the consequence, highlighting the extreme surgical complexity and hostile anatomy.

### Study Limitations

A primary limitation is the retrospective nature of the study that introduces selection bias, since the choice of bailout technique was left to the discretion of the attending surgeon rather than a defined protocol. Furthermore, the treatment protocol of acute cholecystitis was different among participating centers, with some centers still treat acute cholecystitis with antibiotic therapy and delayed cholecystectomy, providing another source of selection bias. Finally the BOLAC study had a limited 30-day follow-up period. While sufficient for capturing immediate postoperative morbidity, this timeframe was insufficient to identify long-term complications or late-onset issues specifically associated with subtotal techniques, such as recurrent cholecystitis in the remaining gallbladder stump or late biliary strictures. Additionally, because the primary objective of the BOLAC study was to assess the overall incidence and predictors of the decision to deviate from standard laparoscopic completion, we evaluated bailout procedures as a collective entity, according to existing literature. Consequently, the study was not statistically powered to perform isolated multivariable risk-factor profiling for individual sub-techniques, though unadjusted outcome trends for each approach are provided for transparency in the supplementary data. On the other hand the study is strengthened by its multicenter design and the large sample size, providing a realistic "snapshot" of emergency surgical practice in northern Italy.

## Conclusion

This large multicenter analysis demonstrates that bailout procedures during laparoscopic cholecystectomy for acute cholecystitis are required in approximately one in 1 in 10 patients, confirming their role as a relatively frequent component of real-world emergency surgical practice. Their occurrence is strongly associated with identifiable preoperative risk factors such as the presence of intramural abscess and peritoneal collection at preoperative imaging as well as male sex and delayed surgical timing beyond seven days from symptom onset. These findings highlight the importance of early diagnosis, timely surgical intervention, and meticulous preoperative imaging in anticipating operative difficulty. Recognizing patients at higher risk for bailout may support better operative planning, resource allocation, and informed decision-making, with the goal of balancing the protective intent of bailout strategies against their associated morbidity.

## Supplementary Information

Below is the link to the electronic supplementary material.

Supplementary file1 (DOCX 24 KB)

Supplementary file2 (DOCX 21 KB)
