# Supplementary material for: Risk factors and outcomes of bailout procedures during laparoscopic cholecystectomy for acute cholecystitis: the BOLAC study
Source: Surg Endosc. 2026 Jul 15;40(10):8485–96. doi: 10.1007/s00464-026-13040-9 (PMC13633154; doi:10.1007/s00464-026-13040-9)
Supplement: Supplementary file 1 [file 464_2026_13040_MOESM1_ESM.docx]

Supplementary Table S1 Detailed patients’ characteristics

|  |  | Overall |  | Laparopscopic cholecystectomy |  | Laparoscopic subtotal cholecystectomy |  | Open cholecystectomy |  | Open subtotal cholecystectomy |  | p |
| --- | --- | --- | --- | --- | --- | --- | --- | --- | --- | --- | --- | --- |
| n |  | 1805 |  | 1629 |  | 68 |  | 85 |  | 23 |  |  |
| age (mean (SD)) |  | 62.20 (16.24) |  | 61.57 (16.38) |  | 63.90 (12.37) |  | 70.62 (13.45) |  | 70.52 (14.94) |  | <0.001 |
| sex M(%) |  | 1033 (57.2) |  | 911 (55.9) |  | 46 (67.6) |  | 59 ( 69.4) |  | 17 ( 73.9) |  | 0.008 |
| BMI (mean (SD)) |  | 27.44 (4.83) |  | 27.39 (4.81) |  | 28.37 (4.54) |  | 27.47 (5.09) |  | 27.75 (5.91) |  | 0.646 |
| Charlson Comorbidity Index (mean (SD)) |  | 2.52 (2.20) |  | 2.42 (2.15) |  | 2.63 (2.25) |  | 3.96 (2.60) |  | 3.78 (2.07) |  | <0.001 |
| septic shock at presentation (mean (SD)) |  | 33 ( 1.8) |  | 28 ( 1.7) |  | 3 ( 4.4) |  | 2 ( 2.4) |  | 0 ( 0.0) |  | 0.363 |
| severe sepsis at presentation (mean (SD)) |  | 150 ( 8.3) |  | 128 ( 7.9) |  | 12 (17.9) |  | 9 ( 10.6) |  | 1 ( 4.3) |  | 0.023 |
| WBC (median [IQR]) |  | 13.00 [10.10, 16.51] |  | 13.00 [10.00, 16.26] |  | 13.05 [9.72, 16.12] |  | 15.20 [11.20, 19.55] |  | 13.81 [11.38, 17.43] |  | 0.008 |
| CRP (mg/L) (median [IQR]) |  | 65.60 [15.12, 190.60] |  | 60.00 [14.00, 175.00] |  | 61.50 [19.46, 198.50] |  | 177.90 [63.20, 257.30] |  | 116.00 [24.90, 291.00] |  | <0.001 |
| PCT (median [IQR]) |  | 0.80 [0.14, 3.07] |  | 0.76 [0.12, 2.78] |  | 2.30 [0.49, 6.30] |  | 1.13 [0.64, 11.30] |  | 10.40 [5.28, 10.85] |  | 0.056 |
| Serum bilirubin (median [IQR]) |  | 1.10 [0.70, 1.98] |  | 1.10 [0.70, 1.98] |  | 1.17 [0.70, 1.89] |  | 1.38 [0.88, 2.12] |  | 1.15 [0.87, 1.55] |  | 0.135 |
| US (%) |  | 1386 (76.8) |  | 1267 (77.8) |  | 44 (64.7) |  | 63 ( 74.1) |  | 12 ( 52.2) |  | 0.002 |
| US Wall thickness (mm) (mean (SD)) |  | 6.17 (2.77) |  | 6.16 (2.81) |  | 5.48 (2.33) |  | 6.79 (2.41) |  | 5.66 (2.36) |  | 0.286 |
| Intramural abscesses (%) |  | 143 ( 8.0) |  | 110 ( 6.8) |  | 15 (22.7) |  | 15 ( 17.9) |  | 3 ( 13.0) |  | <0.001 |
| Peritoneal collection (%) |  | 253 (14.1) |  | 209 (12.9) |  | 22 (33.3) |  | 15 ( 17.6) |  | 7 ( 30.4) |  | <0.001 |
| CT scan (%) |  | 774 (43.0) |  | 671 (41.3) |  | 41 (60.3) |  | 46 ( 54.1) |  | 16 ( 69.6) |  | <0.001 |
| Preoperative second level imaging for suspected CBD stones (%) |  | 339 (18.8) |  | 299 (18.4) |  | 17 (25.0) |  | 17 ( 20.0) |  | 6 ( 26.1) |  | 0.424 |
| Preoperative second level imaging performed (EUS/MRCP) (%) | EUS | 169 (48.3) |  | 150 (48.4) |  | 10 (58.8) |  | 4 ( 23.5) |  | 5 ( 83.3) |  | 0.049 |
|  | MRCP | 181 (51.7) |  | 160 (51.6) |  | 7 (41.2) |  | 13 ( 76.5) |  | 1 ( 16.7) |  |  |
| Preoperative ERCP (%) |  | 171 ( 9.5) |  | 148 ( 9.1) |  | 14 (20.6) |  | 5 ( 5.9) |  | 4 ( 17.4) |  | 0.005 |
| Preoperative percutaneous cholecystostomy (%) |  | 32 ( 1.8) |  | 24 ( 1.5) |  | 3 ( 4.4) |  | 3 ( 3.5) |  | 2 ( 8.7) |  | 0.010 |
| Time of the day (%) | afternoon | 1028 (57.0) |  | 917 ( 56.4) |  | 40 (58.8) |  | 56 (65.9) |  | 15 (65.2) |  | 0.011 |
|  | morning | 645 (35.8) |  | 601 ( 37.0) |  | 21 (30.9) |  | 19 (22.4) |  | 4 (17.4) |  |  |
|  | night | 129 ( 7.2) |  | 108 ( 6.6) |  | 7 (10.3) |  | 10 (11.8) |  | 4 (17.4) |  |  |
| Calendar period (%) | week | 1454 (81.2) |  | 1320 ( 81.7) |  | 56 (83.6) |  | 60 (70.6) |  | 18 (78.3) |  | 0.027 |
|  | weekend | 311 (17.4) |  | 276 ( 17.1) |  | 8 (11.9) |  | 23 (27.1) |  | 4 (17.4) |  |  |
|  | holiday | 25 ( 1.4) |  | 19 ( 1.2) |  | 3 ( 4.5) |  | 2 ( 2.4) |  | 1 ( 4.3) |  |  |
| Timinig from admission to surgery (days) (mean (SD)) |  | 2.96 (4.70) |  | 2.88 (4.60) |  | 4.16 (5.17) |  | 3.34 (5.93) |  | 3.78 (5.27) |  | 0.102 |
| Reason for bailout procedure (%) | CVS not achieved | 85 (54.5) |  | 0 ( 0.0) |  | 48 (73.8) |  | 26 (37.1) |  | 10 (50.0) |  | 0.010 |
|  | Adhesions | 46 (29.5) |  | 0 ( 0.0) |  | 14 (21.5) |  | 24 (34.3) |  | 8 (40.0) |  |  |
|  | Bleeding | 9 ( 5.8) |  | 0 ( 0.0) |  | 1 ( 1.5) |  | 8 (11.4) |  | 0 ( 0.0) |  |  |
|  | CBD injury / Bile leak | 2 ( 1.3) |  | 0 ( 0.0) |  | 0 ( 0.0) |  | 2 ( 2.9) |  | 0 ( 0.0) |  |  |
|  | Other | 14 ( 9.0) |  | 0 ( 0.0) |  | 2 ( 3.1) |  | 10 (14.3) |  | 2 (10.0) |  |  |
| Sub-speciality of the operator | Acute care | 940 (52.1) |  | 844 ( 51.8) |  | 30 (44.1) |  | 53 (62.4) |  | 13 (56.5) |  | 0.211 |
|  | Colorectal | 178 ( 9.9) |  | 164 ( 10.1) |  | 10 (14.7) |  | 3 ( 3.5) |  | 1 ( 4.3) |  |  |
|  | HPB | 228 (12.6) |  | 212 ( 13.0) |  | 7 (10.3) |  | 6 ( 7.1) |  | 3 (13.0) |  |  |
|  | other | 459 (25.4) |  | 409 ( 25.1) |  | 21 (30.9) |  | 23 (27.1) |  | 6 (26.1) |  |  |
| Age of the operator (mean (SD)) |  | 47.22 (9.44) |  | 47.05 (9.30) |  | 49.88 (10.12) |  | 48.05 (10.97) |  | 48.09 (10.28) |  | 0.079 |
| ICG adopted(%) |  | 376 (20.8) |  | 345 ( 21.2) |  | 21 (30.9) |  | 6 ( 7.1) |  | 4 (17.4) |  | 0.003 |
