# Supplementary material for: Risk factors and outcomes of bailout procedures during laparoscopic cholecystectomy for acute cholecystitis: the BOLAC study
Source: Surg Endosc. 2026 Jul 15;40(10):8485–96. doi: 10.1007/s00464-026-13040-9 (PMC13633154; doi:10.1007/s00464-026-13040-9)
Supplement: Supplementary file 2 [file 464_2026_13040_MOESM2_ESM.docx]

Supplementary Table S2 Detailed outcomes by each specific procedures

|  | level |  | Laparoscopic cholecystectomy |  | Laparoscopic subtotal cholecystectomy |  | Open cholecystectomy |  | Open subtotal cholecystectomy |  | p |
| --- | --- | --- | --- | --- | --- | --- | --- | --- | --- | --- | --- |
| n |  |  | 1629 |  | 68 |  | 85 |  | 23 |  |  |
| Mortality (%) |  |  | 3 ( 0.2) |  | 2 ( 2.9) |  | 1 ( 1.2) |  | 0 ( 0.0) |  | <0.001 |
| Morbidity, any (%) |  |  | 398 (24.4) |  | 29 (42.6) |  | 39 (45.9) |  | 5 (21.7) |  | <0.001 |
| Complications grade(%) | 0 |  | 1231 (75.6) |  | 39 ( 57.4) |  | 46 (54.1) |  | 18 ( 78.3) |  | <0.001 |
|  | I |  | 241 (14.8) |  | 8 ( 11.8) |  | 6 ( 7.1) |  | 1 ( 4.3) |  |  |
|  | II |  | 85 ( 5.2) |  | 8 ( 11.8) |  | 14 (16.5) |  | 1 ( 4.3) |  |  |
|  | IIIa |  | 45 ( 2.8) |  | 9 ( 13.2) |  | 15 (17.6) |  | 3 ( 13.0) |  |  |
|  | IIIb |  | 14 ( 0.9) |  | 2 ( 2.9) |  | 1 ( 1.2) |  | 0 ( 0.0) |  |  |
|  | IV |  | 10 ( 0.6) |  | 0 ( 0.0) |  | 2 ( 2.4) |  | 0 ( 0.0) |  |  |
|  | V |  | 3 ( 0.2) |  | 2 ( 2.9) |  | 1 ( 1.2) |  | 0 ( 0.0) |  |  |
| Length of stay (mean (SD)) |  |  | 6.71 (7.04) |  | 12.34 (11.61) |  | 11.89 (9.67) |  | 9.91 (6.51) |  | <0.001 |
| Reoperation (%) |  |  | 14 ( 0.9) |  | 2 ( 2.9) |  | 1 ( 1.2) |  | 1 ( 4.3) |  | 0.135 |
| Severe complications (%) |  |  | 27 ( 1.6) |  | 4 ( 5.9) |  | 4 ( 4.7) |  | 0 ( 0.0) |  | 0.013 |
| Bile leak (%) |  |  | 38 ( 2.3) |  | 10 ( 14.7) |  | 9 (10.6) |  | 0 ( 0.0) |  | <0.001 |
| CBD injury (%) |  |  | 5 ( 0.3) |  | 1 ( 1.5) |  | 5 ( 5.9) |  | 0 ( 0.0) |  | <0.001 |
| Superficial SSI (%) |  |  | 16 ( 1.0) |  | 1 ( 1.5) |  | 4 ( 4.7) |  | 1 ( 4.3) |  | 0.010 |
| Deep SSI (abscess/collection) (%) |  |  | 44 ( 2.7) |  | 7 ( 10.3) |  | 6 ( 7.1) |  | 2 ( 8.7) |  | <0.001 |
| Respiratory infections (%) |  |  | 27 (1.7) |  | 7 (10.3) |  | 6 (7.1) |  | 0.00 (0.00) |  | <0.001 |
| Renal failure (%) |  |  | 21 ( 1.3) |  | 4 ( 5.9) |  | 7 ( 8.2) |  | 1 ( 4.3) |  | <0.001 |
| Urinary infections (%) |  |  | 7 ( 0.4) |  | 0 ( 0.0) |  | 1 ( 1.2) |  | 0 ( 0.0) |  | 0.694 |
| Cardio complications (%) |  |  | 29 ( 1.8) |  | 4 ( 5.9) |  | 5 ( 5.9) |  | 1 ( 4.3) |  | 0.009 |
| Readmission within 30 days (%) |  |  | 37 ( 2.3) |  | 5 ( 7.4) |  | 6 ( 7.1) |  | 1 ( 4.3) |  | 0.005 |
